# The effect of suramin on inhibiting fibroblast proliferation and preventing epidural fibrosis after laminectomy in rats

**DOI:** 10.1186/s13018-016-0443-5

**Published:** 2016-10-01

**Authors:** Jihang Dai, Xiaolei Li, Lianqi Yan, Hui Chen, Jun He, Shuguang Wang, Jingcheng Wang, Yu Sun

**Affiliations:** Department of Orthopedics, Clinical Medical College of Yangzhou University, Orthopaedic Institute, Subei People’s Hospital of Jiangsu Province, Yangzhou, 225001 China

**Keywords:** Suramin, Fibroblasts proliferation, Epidural fibrosis

## Abstract

**Background:**

Epidural fibrosis often causes serious complications in patients after lumbar laminectomy and discectomy and is associated with the proliferation of fibroblasts. Suramin is known to have an obvious inhibitory effect on the coactions of many growth factors and their receptors, but little was previously known about the effect of suramin on fibroblast proliferation and the progress of epidural fibrosis.

**Methods:**

We illustrated the effect of suramin on cultured fibroblasts of rats with different concentrations (0, 200, 400, 600 mg/l). The proliferation of suramin-treated fibroblasts was evaluated by CCK-8 and western blot analysis. Additionally, in a rat model of laminectomy, different concentrations of suramin (100, 200, and 300 mg/ml) and saline were applied to the laminectomy sites locally. The effect of suramin on preventing epidural fibrosis was detected by the Rydell classification, hydroxyproline content, histological analysis, and collagen density analyses.

**Results:**

The results of CCK-8 shown that suramin could significantly inhibit fibroblasts proliferation in a dose-dependent manner. The western blotting shown that the expression levels of the cell proliferation markers such as cyclin D1, cyclin E, and PCNA were down-regulated. Moreover, in a rat model, we found that suramin could reduce epidural fibrosis as well as inhibit fibroblast proliferation, and 300 mg/ml suramin had better effect.

**Conclusions:**

Topical application of suramin could reduce epidural fibrosis after laminectomy, and the application of suramin could inhibit the proliferation of fibroblasts in rats. This study indicates that suramin is a potent antifibrotic agent that may have therapeutic potential for patients with epidural fibrosis.

## Background

Epidural fibrosis following lumbar laminectomy and discectomy is a common outcome and remains a challenging clinical problem for surgeons [1, 2]. It may result in unfavorable clinical outcome and contribute to more complications in revision spine surgery, such as dural tears, nerve root injury, and bleeding. Although the definite mechanism of fibrosis formation is still unclear, some reports have shown that fibroblast proliferation and the succeeding release of extracellular matrix were the main reasons for epidural fibrosis [3].

At present, the process of epidural fibrosis cannot be effectively prevented. In order to control epidural fibrosis, many therapeutic strategies have been sought to reduce tissue damage and avoid adverse complication [4, 5]. For example, various agents or mechanical barriers have been used to prevent epidural fibrosis both in animal models and humans, including Adcon-L, autologous fat grafts, fibrinolytic agents, and polytetrafluoroethylene membrane [6–8]. However, all of these techniques are not without complications.

Suramin, a polyanionic drug, specifically inhibits many growth factors. In clinical practice, suramin has been commonly used to treat trypanosomiasis and onchocerciasis by inhibiting DNA polymerases and reverse transcriptase [9]. Recently, suramin has been demonstrated to be effective in reducing peritoneal fibrosis [10], chronic renal fibrosis [11], and the fibrosis of proliferative eye disease [12]. Also, it was found that suramin could inhibit cell proliferation [13–15], which implicated that suramin might be useful in prevention of epidural fibrosis through inhibiting the proliferation of fibroblasts.

Therefore, we designed the study to explore the possible preventive effect of local application of suramin in preventing and reducing epidural fibrosis in rats after laminectomy. Additionally, we investigated suramin’s effects on fibroblast proliferation in vitro at different concentrations. Our results may be helpful for future human trials and clinical applications for treating epidural fibrosis.

## Methods

### Cell culture and treatment

A primary fibroblast cell line was established from epidural scar tissue isolated from rats that underwent laminectomies 4 weeks later [16]. Fibroblasts were grown in Dulbecco’s modified Eagle’s medium (DMEM; Gibco, Grand Island, NY) with 15 % fetal bovine serum, 0.1 U/l penicillin, and 50 μg/ml streptomycin (PS; Thermo, Rockford, IL) at 37 °C in 5 % CO_2_. Medium was changed every 3 days. Cells between passages 4 and 7 were used for all experiments. After reaching approximately 60–70 % confluence, the cells were cultured in fresh medium without fetal bovine serum overnight and then treated with suramin of different concentrations and combinations of all substances.

### Cell viability

Cell Counting Kit-8 (CCK-8; Dojindo, Tokyo, Japan) was used to detect the cell viability. Fibroblasts at passages 4 and 7 were seeded in four replicates in 96-well plates (100 ml, 2 × 10^3^/well) overnight. Then, fibroblasts were treated with suramin of different concentrations (0, 200, 400, 600 mg/l). Forty-eight hours later, the cells were further incubated with 10 μl CCK-8 solution for 2 h. Cells that stained positively with CCK-8 were considered viable. Cell survival rate was calculated according to the reference manual.

### Western blotting analyses

After received various treatments, the fibroblasts were collected. In order to carry out western blot analysis, the cells should be first treated with RIPA buffer on ice. Following sonicated and centrifuged in sequence, the proteins were collected for western blot analysis. BCA Protein Assay Kit was used to measure protein concentrations. The proteins were separated on a 6–12 % SDS-polyacrylamide gel electrophoresis and then transferred for 1.5 h at 200 mA to polyvinylidene difluoride membranes (Millipore, Bedford, MA) on ice. Then the PVDF membranes were blocked with 5 % skimmed milk in tris-buffered saline and Tween 20 for 2 h at room temperature and incubated with primary antibodies at 4 °C overnight. The primary antibodies were anti-cyclinD1, anti-cyclinE, anti- polyclonal proliferating cell nuclear antigen (PCNA), and anti-β-actin antibodies (Cell Signaling Technology, USA). Next day, after washing, the membranes were incubated with anti-rabbit/mouse antibodies (Cell Signaling Technology, USA) for 1.5 h at room temperature. Then, we washed the membranes for three times. At last, the membranes were exposed using the ECL system (Millipore, Bedford, USA).

### Animals

This study uses 48 adult, healthy, Sprague-Dawley rats. All these white rats, whose mean weight was 260 g, were purchased from Yangzhou Laboratory Animal Center, Yangzhou, China. All animals received care in accordance with the principles of Laboratory Animal Care of international recommendation. 48 healthy rats and were divided into four groups (12 rats in each group): suramin (300 mg/ml) group; suramin (200 mg/ml) group; suramin (100 mg/ml) group, and control (saline) group.

### Reagents

Suramin was purchased from Cayman Chemical Co., USA.

### Rat model

The surgery of laminectomy on rat was operated by the steps described before [17]. Before the surgery, each rat was anesthetized using 1 % pentobarbital sodium by intra-peritoneal injection. Then, we shaved the rat around L1 and L2 and sterilized the exposed skin after the shaving. After exposing the fascia and the paraspinal muscles by a midline skin incision, the spinous process and vertebral plate of the L1 and L2 were removed by a rongeur. Then, a complete 5 × 2 mm area of the dura mater was exposed. All rats underwent a total L1 laminectomy.

### Local application of drugs

Following satisfactory hemostasis, cotton pads soaked with suramin in various concentrations of 100, 200, 300 mg/ml, and saline (9 mg/ml) were administered to the exposed laminectomy sites for 5 min. The surrounding tissues were covered by wet gauzes to avoid touching the agent. Then, the suramin-soaked cotton wool was removed and the decorticated areas of laminectomy were irrigated with saline to get rid of the remaining suramin immediately. After the above operations, the wounds were closed in layers.

### Macroscopic assessment of epidural fibrosis

After 4 weeks, six rats were randomly picked from each group for macroscopic evaluation. The surgical sites were reopened through previous operative incision, and the epidural scar fibrosis was evaluated under double-blind trials according to Rydell’s standard [18, 19]: grade 0, little epidural scar tissue without adherence to the dura mater; grade 1, moderate epidural scar tissue with slight adherence to the dura mater; grade 2, moderate epidural scar tissue with tight adherence to the dura mater and dissected with difficulty without disrupting the dura matter; and grade 3, epidural scar tissue was firmly adherent to the dura mater and could not be dissected.

### Determination of hydroxyproline content in epidural scar tissue

After macroscopic observation, the rats were euthanized with a high dose of pentobarbital sodium, and about 5 mg (wet weight) of scar tissue was got from the decorticated areas. The samples were lyophilized, grounded, and hydrolyzed separately. Then, they were neutralized with 2.5 N NaOH by using methyl red as the indicator. One milliliter chloramine-T was added to the hydrolyzed samples as well as four hydroxyproline standards of four known concentrations. Following 20-min incubation at room temperature, the hydroxyproline developer was added to the samples and standards. A spectrophotometer was used to measure the absorbance of the solution at 558 nm, and the calculation of the hydroxyproline content per milligram of scar tissue was based on the standard curve constructed by the serial concentration of commercial hydroxyproline.

### Histological analysis

Six rats were picked randomly from the four groups at 4 weeks postoperatively. Following anesthesia by pentobarbital sodium, a 4 % paraformaldehyde solution intracardially perfused was needed for histological analysis. The entire L1 spine column, including around tissues, was removed. After 4 days soak by 10 % buffered formalin, each specimen was decalcified in ethylenediamine tetraacetic acid (EDTA) and glycerol solution for 30 days to decalcify, then embedded in paraffin. The upright L1 vertebra has made 12 continuous transversal sections of 4 μm from the highest organizations to the grass root. From which of them, six odd sections were stained by means of hematoxylin and eosin (H&E), and the epidural scar adhesion was assessed through a light microscope with the magnification of ×40. At a magnification of ×400, three fields of the laminectomy sites in each section, fibroblast density was calculated. The other six even sections use the method of Trichrome stained by Masson, and the process of collagen tissue proliferation was presented through the ×200 magnified light microscope. Also, Image Pro Plus 6.0 image analysis software was used to determine the optical density value of positively stained collagen.

### Statistical analysis

All of the statistical analyses were performed by SPSS software (version 15.0). The results of data were expressed as mean ± standard deviation value. For all analyses, *P* values <0.05 were considered statistically significant.

## Results

### The effect of suramin on fibroblast proliferation

**Fig. 1:**
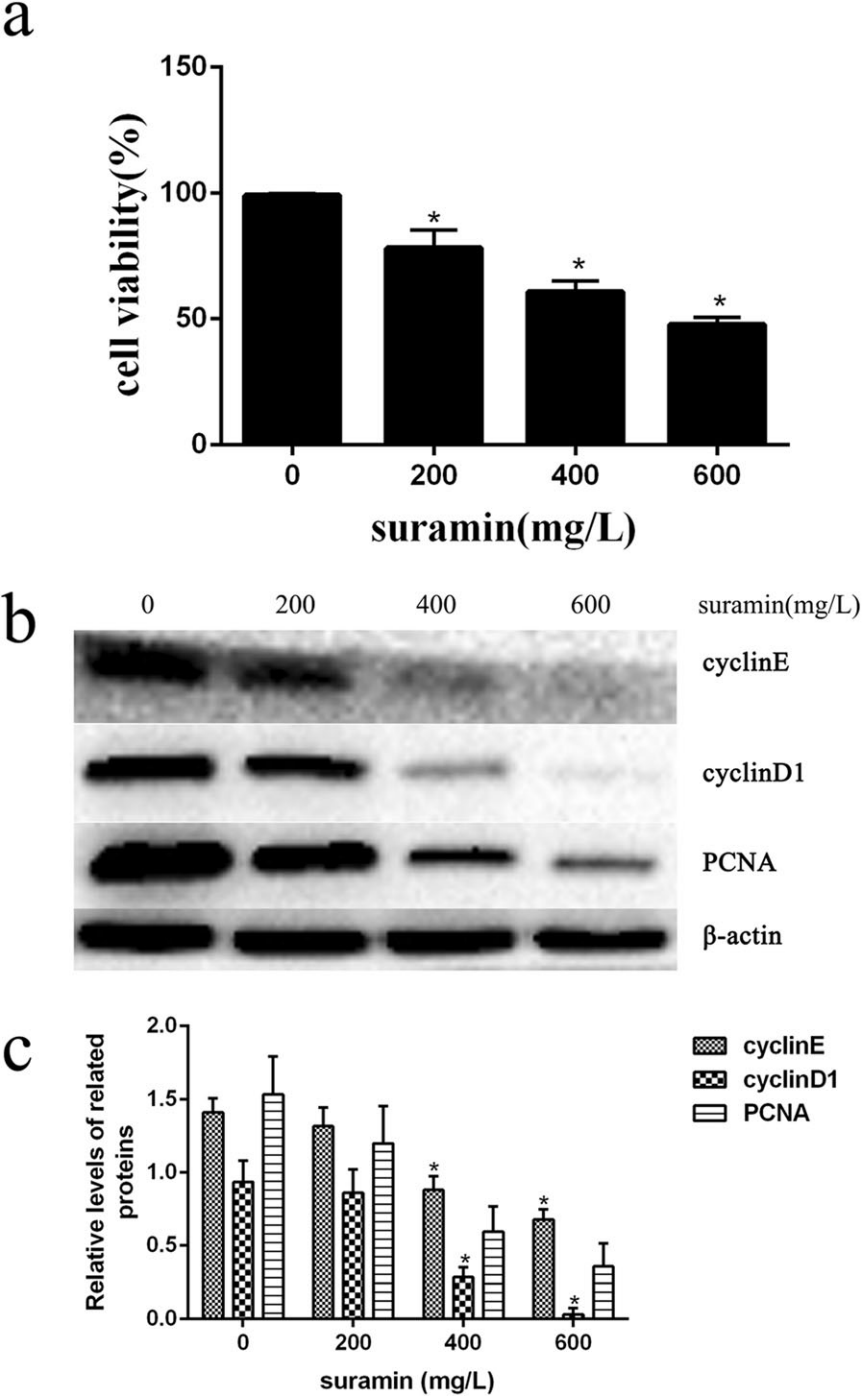
Suramin inhibited fibroblast proliferation. After treating with suramin for 48 h at doses of 0, 200, 400, 600 mg/l, CCK-8 assay (**a**) and western blot assay of the expression of cell proliferation markers (cyclinD1, cyclinE, and PCNA) were used to detect cell proliferation (**b**). The expression of cyclinD1, cyclinE, and PCNA was decreased in a dose-dependent manner. The band intensities for cyclinD1, cyclinE, and PCNA were expressed as a histogram relative to β-actin (**c**). *P < 0.05 versus controls

To clarify the effect of suramin on fibroblasts proliferation, we used suramin at concentrations of 0, 200, 400, and 600 mg/l to treat fibroblasts for 48 h. After the fibroblasts were treated with four known concentrations of suramin, the results of the CCK-8 assay revealed suramin could inhibit fibroblast proliferation and were shown in a dose-dependent manner (Fig. 1a).

We further confirmed the effect of suramin on expression of the cell proliferation markers [20, 21], and the results of western blot shown that suramin could decrease the expressions of cyclin D1, cyclin E, and PCNA (Fig. 1b, c). All these results indicate that suramin could inhibit the proliferation of the fibroblast in a dose-dependent manner.

### Macroscopic evaluation of scar adhesion

The results of macroscopic observation, classified according to Rydell’s classification, suggested that grade 3 epidural adhesions existed in all of the control group rats. Grade 0, 1, and 2 were found in the suramin-treated group (Table 1).

**Table 1:** The degree of epidural scar adhesion according to the Rydell standard

Group	Grade			
	0	1	2	3
Suramin (300 mg/ml)	4	2	0	0
Suramin (200 mg/ml)	3	2	1	0
Suramin (100 mg/ml)	0	0	3	3
Saline (9 mg/ml)	0	0	0	6

### Hydroxyproline content analysis

**Fig. 2:**
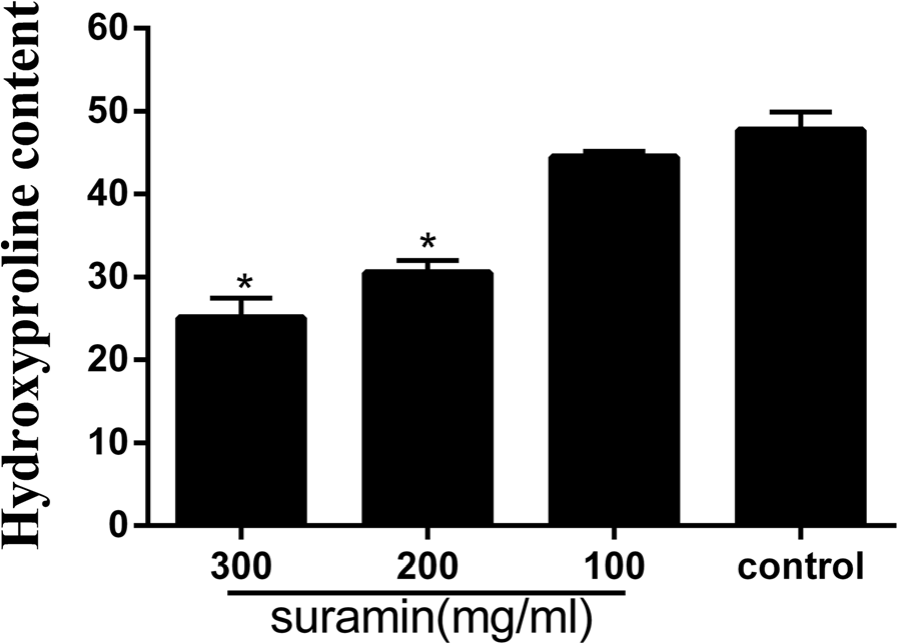
Hydroxyproline contents in epidural scar tissue in suramin-treated groups and control group. Hydroxyproline content was expressed as micrograms per milligrams. *P < 0.05 compares with that in control group

The hydroxyproline contents of each group were shown in Fig. 2. The hydroxyproline contents in 300 and 200 mg/ml suramin groups were less than those in 100 mg/ml suramin group (*P* < 0.05) and in control group (*P* < 0.05). The hydroxyproline content in 300 mg/ml suramin group was also less than in 200 mg/ml suramin group (*P* < 0.05). However, the difference between 100 mg/ml suramin and control group was not significant (*P* = 0.088).

### Effect of suramin on epidural fibrosis in histological analysis

**Fig. 3:**
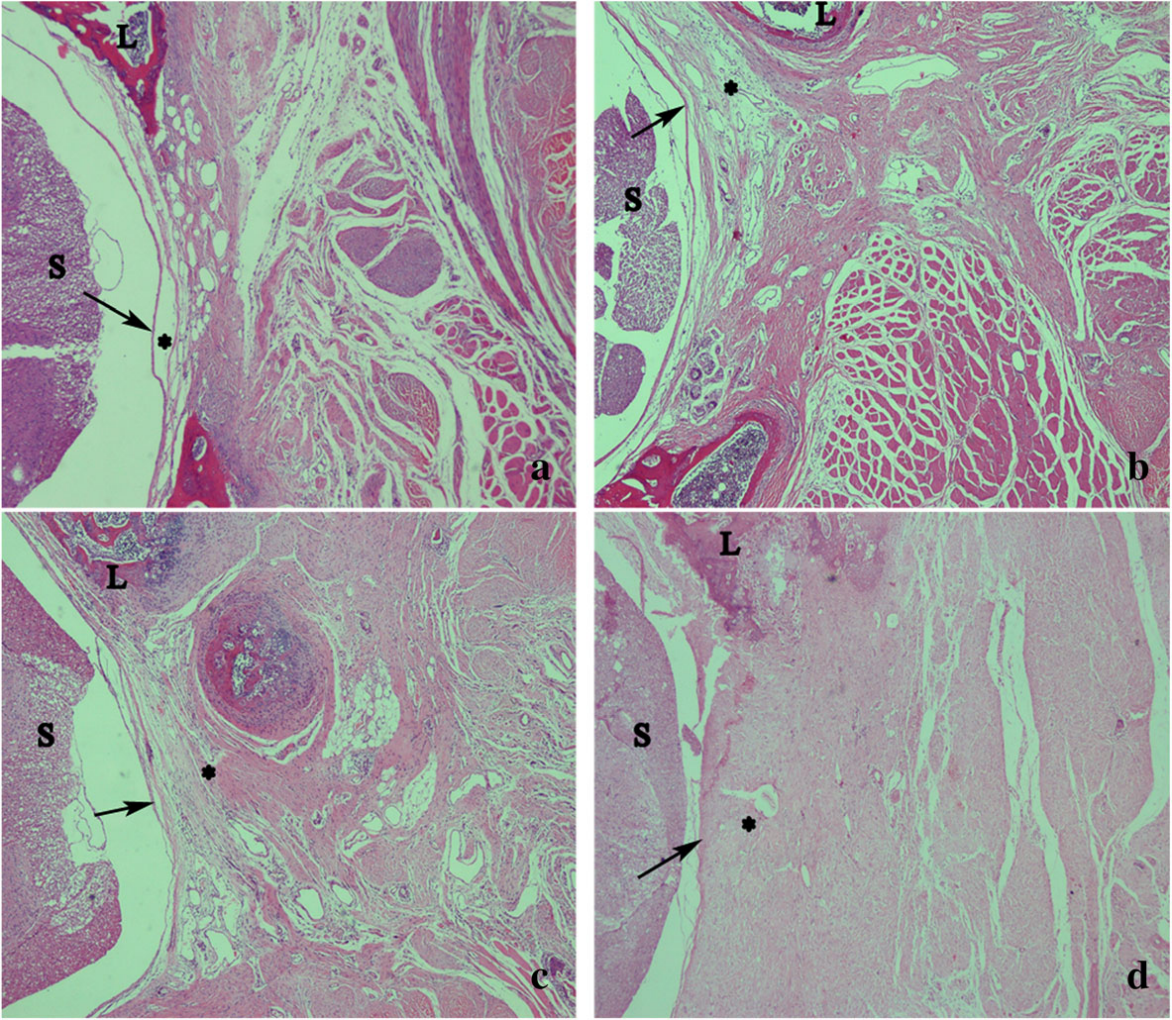
The representative images of the epidural fibrosis issues in each group. Laminectomy sites treated with 100 mg/ml suramin (**c**) and saline (control, **d**) show that dense epidural fibrosis (*asterisk*) firmly adhering to the dura mater (*arrow*). Moderate epidural fibrosis (*asterisk*) slightly adhering to the dura mater were found in 200 mg/ml suramin group (**b**). Thin epidural fibrosis (*asterisk*) with little adherence to the dura mater were found in 300 mg/ml suramin group (**a**). The spinal cord was represented by “*S*” and laminectomy defect was represented by “*L*”. The magnification was ×40

**Fig. 4:**
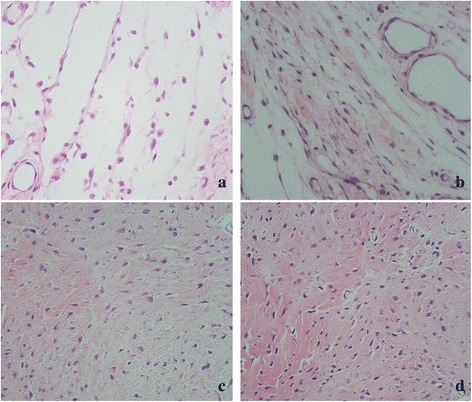
Image of fibroblast in epidural fibrosis tissue of the laminectomy sites treated with suramin of 300 mg/ml (**a**), 200 mg/ml (**b**), 100 mg/ml (**c**), and saline (control, **d**). Note that less fibroblasts were found in the 300 mg/ml suramin group (**a**) and 200 mg/ml suramin group (**b**). However, a large number of fibroblasts were found in 100 mg/ml suramin group (**c**) and control group (**d**). The magnification is ×400

In the laminectomy sites of 100 mg/ml suramin and control group, thick epidural fibrosis with extensive adherence to dura mater was found (Fig. 3c, d), and a particularly large number of fibroblasts were found outside the dura mater (Fig. 4c, d). In 200 mg/ml suramin group, less epidural fibrosis was seen and fibroblasts were decreased around the laminectomy sites compared with all those in control group (Figs. 3b and 4b). What is more, little epidural fibrosis was found in the laminectomy sites of 300 mg/ml suramin group and the number of fibroblasts was markedly reduced (Figs. 3a and 4a).

**Fig. 5:**
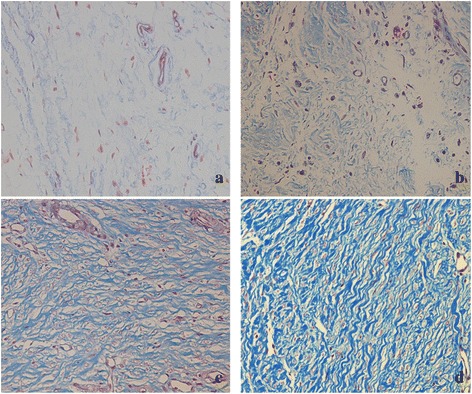
Histological images of collagen tissue hyperplasia in epidural scar tissue of each group. The collagen tissues in the section stained with Masson shown *blue*. There was low density collagen in 300 mg/ml suramin-treated group (**a**), which was significantly lesser than the other groups. Moreover, the density of collagen tissue in 200 mg/ml suramin-treated group (**b**) was also less than the 100 mg/ml suramin-treated group (**c**) and saline group (**d**). The magnification was ×200

As shown in Fig. 5a, b, collagen-tissue hyperplasia was reduced and less epidural fibrosis were found outside the dura mater compared with those of 100 mg/ml treated group and control group. Particularly, the collagen-tissue hyperplasia in 300 mg/ml suramin-treated group was markedly reduced (Fig. 5a).

### Effect of suramin on fibroblasts counting in histological analysis

**Fig. 6:**
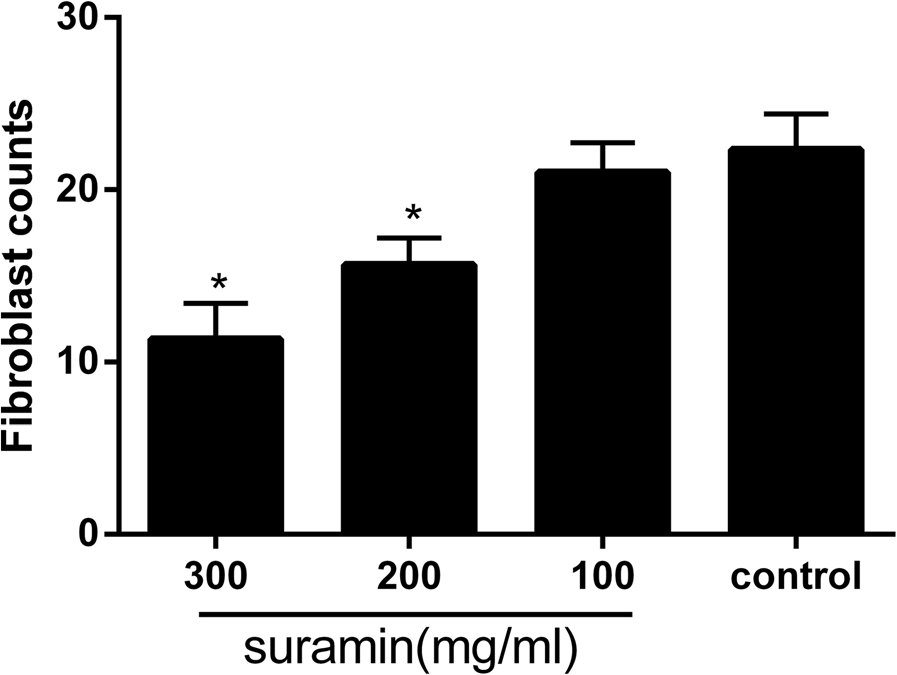
The fibroblast counts in the four groups of epidural scar tissue. Fibroblast counting was shown as the number per counting area. *P < 0.05 versus the control group

As shown in Fig. 6, fibroblasts counting in epidural fibrosis tissue in the 300 mg/ml suramin group was markedly decreased compared with the control group (*P* < 0.05). Besides, fibroblasts counting in the 200 mg/ml suramin group was also less than the control group (*P* < 0.05). However, the difference between 100 mg/ml suramin group and control group had no statistical significance (*P* = 0.15).

### Effect of suramin on collagen density in histological analysis

**Fig. 7:**
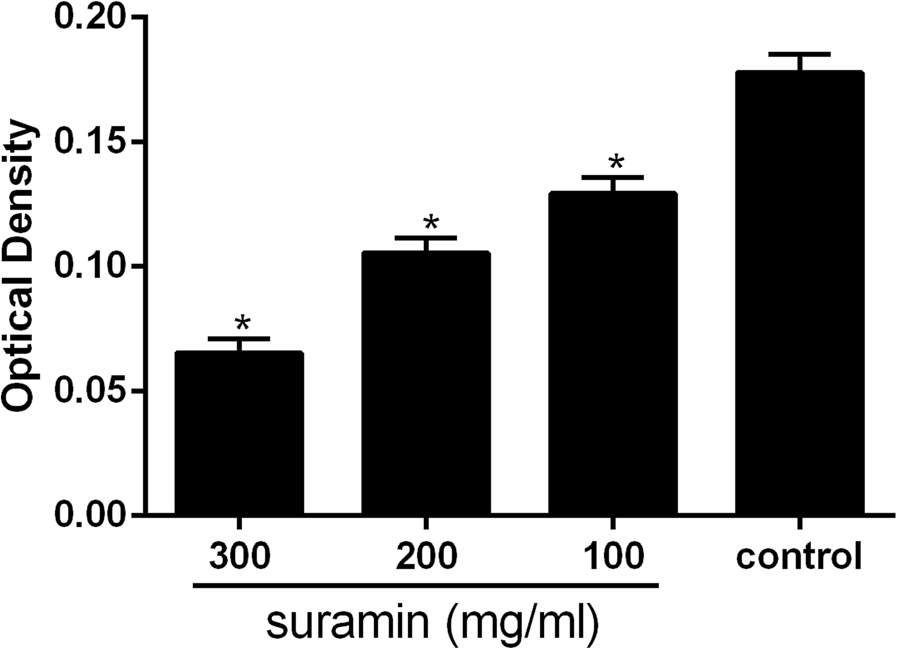
The collagen optical density of four groups. *P < 0.05 versus the control group

As shown in Fig. 7, the optical density value of collagen tissue in the four groups was shown in a dose-dependent manner. The collagen density was low in the epidural scar tissue of the suramin-treated groups compared with that of the control group (*P* < 0.05).

## Discussion

The dense and thick epidural fibrosis that developed after laminectomy often results in serious negative effects, which is characterized by severe, chronic, and disabling nerve radicular or low back pain after laminectomy. Several factors such as epidural hematoma, fat destruction, and paraspinal muscular fiber invasion influence the formation of epidural fibrosis [22]. Moreover, fibroblasts produce a large amount of collagen and extracellular matrix components in laminectomy areas, which promote the formation of epidural fibrosis. In the past few decades, various biological and synthetic materials were used to reduce the formation of fibrosis. Besides, some drugs such as chemotherapy drugs and nonsteroidal anti-inflammatory drugs were proved to be useful in preventing the epidural fibrosis [23, 24].

As an anti-parasitic drug, suramin has a definite effect in treatment of trypanosomiasis and onchocerciasis by inhibiting reverse transcriptase. It is also used to treat selected malignancies, metastatic diseases, and AIDS [25]. Besides, it is well known that suramin can inhibit the activity of many growth factors and cytokine receptors such as TGF-β, PDGF, and bFGF, as well as some inflammatory cytokines. Of those factors, TGF-β and PDGF are especially important on fibroblasts activity in adhesion formation and wound healing process, which not only increase the proliferation of fibroblasts but also enhance the secretion of extracellular matrix components [26].

The study shown that suramin could inhibit fibroblast proliferation and reduce epidural fibrosis after laminectomy in rats. In vitro, we tested the effect of suramin on fibroblast proliferation from epidural scar tissues. From the CCK-8 analysis (Fig. 1a), we found that suramin had a remarkable inhibition of fibroblast proliferation with a concentration-dependent manner. Furthermore, western blot analysis (Fig. 1b, c) shown that suramin could decrease the expression of cell proliferation markers such as cyclin D1, cyclin E, and PCNA. Consistent with the previous studies, our study shown that different concentrations of suramin could inhibit fibroblast proliferation.

In vivo, we investigated the anti-fibrosis effect of suramin on preventing the formation of epidural fibrosis. The concentration of topically applied suramin chosen was based on the previous studies [12, 27]. Macroscopic evaluation and histological observation (Figs. 3, 4) showed that 300 and 200 mg/ml suramin could reduce epidural fibrosis as well as inhibit fibroblast proliferation after laminectomy. However, 100 mg/ml failed. Moreover, the Rydell classification in 300 mg/ml suramin group was mainly defined at grades 0 to 1, which was much better than those of other groups (Table 1). Besides, the collagen density (Fig. 5) and hydroxyproline content (Fig. 2) were also significantly decreased in 300 and 200 mg/ml suramin group. What is more, all these parameters indicated that 300 mg/ml suramin had better effect in reducing epidural fibrosis compared with other groups.

Based on the previous reports and our study this time, we could explain schematically the effect of suramin on reducing epidural fibrosis. Suramin could inhibit the combination between the multiple cytokines or growth factors with their receptors. And in the development of epidural fibrosis, the production of numerous cytokines or growth factors and subsequent activation of their receptors were increased. Once the multiple cytokines or growth factors were inhibited, the number of fibroblast and the formation of epidural fibrosis of the laminectomy areas accordingly decreased, which accorded with our present results. However, the toxicity of suramin via local absorption is still unknown, and some reports shown that local application of suramin could result some adverse reactions [28]. Therefore, the surface area of application should be limited, and the safety margins determined. What is more, the definite mechanism of suramin on inhibiting proliferation of fibroblasts and reducing epidural scar adhesion was still unclear, and further research should be carried out along this line of thought.

## Conclusions

In summary, our findings firstly demonstrated that suramin could inhibit proliferation of fibroblasts and reduce epidural fibrosis after laminectomy in rats. There were no wound infection, healing disorder of the skin, and other side effects in any rat. It might provide a new target for the treatment of epidural fibrosis. However, the potential complication, the format used, and the long-term effects should be kept in mind prior to clinical application.

## Abbreviation

HE: Hematoxylin and eosin
